# Supplementary material for: Characterization and Evaluation of Taihe Black-Boned Silky Fowl Eggs Based on Physical Properties, Nutritive Values, and Flavor Profiles
Source: Foods. 2024 Oct 18;13(20):3308. doi: 10.3390/foods13203308 (PMC11508104; doi:10.3390/foods13203308)
Supplement: Supplementary file 1 [file foods-13-03308-s001.zip › foods-3248010-SI.pdf]

Table S1. The content of fatty acids in Hy-line Brown, Taihe and crossbred black-boned silky fowl eggs.

Units: mg/g

Fat Acids	TS	CB	HL
C8:0	0.00062 ± 0.000049	0.00060 ± 0.000055	0.00069 ± 0.00011
C10:0	0.0038 ± 0.00026	0.0035 ± 0.00023	0.0035 ± 0.00024
C11:0	0.0016 ± 0.000090	0.0015 ± 0.000079	0.0015 ± 0.00014
C12:0	0.0076 ± 0.0013	0.0069 ± 0.0025	0.015 ± 0.0027
C13:0	0.0022 ± 0.00012	0.0021 ± 0.00015	0.0024 ± 0.00016
C14:0	0.67 ± 0.060	0.59 ± 0.11	0.70 ± 0.10
C14:1N5	0.19 ± 0.025	0.15 ± 0.029	0.25 ± 0.038
C15:0	0.11 ± 0.0076	0.11 ± 0.020	0.15 ± 0.018
C16:0	55.25 ± 3.69	51.29 ± 8.04	57.26 ± 7.02
C16:1N7	6.54 ± 0.95	5.27 ± 0.96	9.34 ± 1.68
C17:0	0.30 ± 0.027	0.32 ± 0.068	0.28 ± 0.038
C17:1N7	0.18 ± 0.012	0.17 ± 0.028	0.20 ± 0.028
C18:0	19.99 ± 1.11	17.40 ± 3.75	16.97 ± 2.59
C18:1TN9	0.23 ± 0.012	0.21 ± 0.033	0.25 ± 0.039
C18:1N9	80.52 ± 3.53	75.50 ± 11.09	83.53 ± 10.92
C18:2TTN6	0.043 ± 0.0036	0.036 ± 0.0045	0.048 ± 0.0084
C18:2N6	30.92 ± 2.20	34.48 ± 6.77	30.77 ± 4.32
C18:3N6	0.24 ± 0.019	0.26 ± 0.048	0.28 ± 0.029
C18:3N3	1.05 ± 0.11	1.07 ± 0.20	0.70 ± 0.091
C20:0	0.077 ± 0.0043	0.064 ± 0.010	0.069 ± 0.0070
C20:1N9	0.45 ± 0.0051	0.44 ± 0.091	0.49 ± 0.075
C20:2N6	0.42 ± 0.0025	0.38 ± 0.066	0.44 ± 0.053
C21:0	0.019 ± 0.0028	0.015 ± 0.0046	0.021 ± 0.0038
C20:3N6	0.35 ± 0.045	0.28 ± 0.033	0.34 ± 0.031
C20:4N6	3.99 ± 0.38	4.04 ± 0.93	3.61 ± 0.56
C20:3N3	0.033 ± 0.0028	0.030 ± 0.0052	0.022 ± 0.0026
C22:0	0.010 ± 0.00052	0.0090 ± 0.0012	0.0078 ± 0.00061
C20:5N3	0.038 ± 0.0046	0.030 ± 0.0052	0.025 ± 0.0020
C22:1N9	0.0050 ± 0.00058	0.0055 ± 0.0011	0.0048 ± 0.00085
C22:4N6	0.37 ± 0.032	0.43 ± 0.081	0.39 ± 0.048
C22:5N6	1.03 ± 0.14	1.10 ± 0.26	1.69 ± 0.26
C24:0	0.0097 ± 0.00032	0.0080 ± 0.00061	0.0076 ± 0.00062
C22:5N3	0.25 ± 0.036	0.25 ± 0.045	0.19 ± 0.026
C24:1N9	1.58 ± 0.14	1.58 ± 0.34	0.98 ± 0.12
C22:6N3	0.45 ± 0.036	0.45 ± 0.085	0.30 ± 0.030

All results are presented as mean values (n = 8) ± standard error. TS, Taihe black-boned silky fowl; CB, crossbred black-boned silky fowl; HL, Hy-line Brown,

Table S2. Thresholds of flavor compounds and odor characteristics in Hy-line Brown, Taihe and crossbred black-boned silky fowl eggs.

Name	TS	CB	HL	Odor Threshold (ppm)	Odor
(R)-(-)-4-Methylhexanoic acid	0.03981±0.04044	0.07026±0.03658	0.01594±0.01338	-	Fatty
Butanoic acid, 3-methyl-	3.90126±6.50304	0.23692±0.26128	7.55584±8.31866	0.49	Sour odor
Propanoic acid, 2-methyl-	1.23268±2.03609	0.09129±0.11476	2.42348±2.50819	6.5505	Sour yogurt smell
Heptanoic acid	0.02495±0.01571	0.03236±0.02493	0.01497±0.02864	0.64	Rancid sourness
Pentanoic acid, 4-methyl-	0.01494±0.01183	0.01113±0.00727	0.02251±0.01817	0.81	Spicy cheese flavor
Pentanoic acid	0.15433±0.108	0.12108±0.05034	0.06609±0.08277	11	Putrid odor
Butanoic acid, 2-methyl-	1.44546±1.90882	0.10256±0.06077	2.46105±2.46331	2.2	Spicy acidic goat cheese aroma
1-Butanol	0.87069±0.81865	1.27205±0.77916	0.28349±0.2283	0.59	Strong alcohol smell
1-Hexanol	0.73861±0.36527	0.38745±0.183	0.56220±0.35617	0.0056	Mild sweetness
1-Hexanol, 3-methyl-	0.02131±0.02822	0.00467±0.00405	0.04813±0.05064	-	-
1-Octen-3-ol	1.52238±1.16046	2.04264±0.56988	0.65559±0.41206	0.0015	Fungal, raw chicken smell
1-Pentanol, 4-methyl-	0.26183±0.21762	0.48742±0.12751	0.09581±0.08636	2.5	Nutty flavor
Ethanol	27.55879±25.66748	17.74041±17.08186	46.21876±7.3558	950	Alcohol smell
Ethanol, 2-butoxy-	1.27422±1.19045	0.51383±0.14212	0.19052±0.26269	0.88	Slightly sour taste
1-Heptanol	0.12656±0.08929	0.07985±0.03033	0.04988±0.03471	0.0048	Faint alcohol aroma

1-Pentanol	0.45862±0.39532	0.54129±0.18428	0.15381±0.12261	0.1502	Pleasant
Isopropyl Alcohol	0.99204±0.71224	3.36080±1.93383	0.68261±0.82304	9.7879	Alcohol smell
E-2-Tetradecen-1-ol	0.48209±0.73711	0.03084±0.02847	0.44147±0.77517	-	-
2-Hexanol	0.03269±0.01828	0.06237±0.03248	0.12468±0.09834	1.5082	Fruit wine aroma
2-Propanol, 1-methoxy-	0.95179±0.81318	5.60052±4.37966	0.32694±0.35167	4	Sweet ether-like odor
2-Heptenal, (Z)-	0.06371±0.06564	0.02753±0.01395	0.02082±0.01358	-	-
Benzaldehyde	0.56489±0.32566	0.64619±0.25598	0.23764±0.2376	0.75	Almond flavor
Undecane	1.95466±1.32536	3.26168±1.4062	0.65358±0.47626	0.62	-
Heneicosane	0.05316±0.05193	0.68759±0.38686	0.02280±0.02696	-	Waxy
Octane, 3,5-dimethyl-	0.34526±0.27548	0.23313±0.07976	0.09785±0.19154	-	-
Tridecane	4.28631±3.91951	6.49858±5.29069	2.52530±4.48523	-	-
Decane, 5-methyl-	0.13791±0.11781	0.09812±0.04953	0.02328±0.04531	-	-
1H-3a,7-Methanoazulene, 2,3,4,7,8,8a-hexahydro-3,6,8,8-tetramethyl-	0.03223±0.02150	0.00891±0.0065	0.10300±0.08110	-	Woody
Decane, 2,6,6-trimethyl-	10.39906±5.59838	10.96555±5.87301	5.47147±5.58389	-	-
Bicyclo[3.1.0]hex-2-ene, 2-methyl-	0.05971±0.04038	0.02236±0.00944	0.16418±0.18976	-	Woody

5-(1-methylethyl)-					
2,4-Dimethyl-1-heptene	0.21563±0.19583	0.06640±0.05274	0.03611±0.06079	-	-
Octane, 2-methyl-	1.31097±1.52546	0.53230±0.37683	0.05744±0.07001	-	-
cis-Thujopsene	0.00363±0.00519	0.00099±0.00047	0.02542±0.02144	-	-
Humulene	0.00528±0.00479	0.00639±0.00425	0.01587±0.01337	0.16	Woody
Undecane, 3-methyl-	0.13776±0.14403	0.1215±0.11809	0.08122±0.09212	-	-
Bis(2-chloro-1-methylethyl) ether	0.17844±0.14194	0.33895±0.1434	0.08539±0.07508	0.3	-
1, 2-Dichloropropane	0.46392±0.39382	1.63089±0.61759	0.64989±0.7026	0.1	Sweet taste
Ethane, 1,1,2,2-tetrachloro-	0.03596±0.04626	0.15569±0.09665	0.05649±0.05848	-	-
Ethane, 1,1,2-trichloro-	0.03345±0.04841	0.0645±0.03993	0.03179±0.02747	-	-
Propane, 1,2,3-trichloro-	0.04426±0.0429	0.10397±0.0963	0.03505±0.02857	-	-
Methanesulfonyl chloride	2.31760±2.25626	3.75489±1.59089	0.68589±0.49404	-	-
Tetrachloroethylene	0.01010±0.01262	0.08189±0.03517	0.02369±0.02422	0.77	Chloroform-like smell
1, 4-Dichlorobenzene	0.01645±0.01589	0.45343±0.21536	0.00999±0.01039	-	Aromatic
Benzene, 1,2,4-trimethyl-	4.89463±5.20253	2.76104±1.04985	0.83408±1.20839	0.12	Aromatic
Benzene, 1-ethyl-2,4-dimethyl-	0.72419±0.72283	0.43009±0.10258	0.16836±0.23018	-	-
Benzene	0.20154±0.17877	0.61545±0.40801	0.15934±0.20607	2.7	Paint thinner-like smell

Benzene, 1-ethyl-3-methyl-	0.98853±1.61932	0.88956±0.9522	0.06394±0.06371	0.018	-
Benzene, 1,3-dimethyl-	0.54299±0.43911	0.51749±0.2311	0.11065±0.14686	0.041	Aromatic
Benzene, propyl-	0.37108±0.38282	0.32294±0.21596	0.06274±0.08514	0.0038	Moth-ball like
Benzene, 1,2,3-trimethyl-	2.81183±2.33554	1.43519±0.48602	0.68967±0.85903	0.032	-
Benzeneacetic acid	0.02225±0.01525	0.01973±0.01569	0.02712±0.0452	-	-
Benzene, 4-ethyl-1,2-dimethyl-	0.48533±0.51423	0.16503±0.11306	0.08830±0.12833	-	-
Benzene, 1,3-bis(1,1-dimethylethyl)-	0.13473±0.37367	0.06265±0.1584	0.00135±0.00158	-	-
Benzene, 1-ethyl-2-methyl-	1.11931±1.11047	0.85656±0.50462	0.16776±0.25548	0.074	-
Benzene, chloro-	0.02262±0.02858	0.11441±0.08123	0.05170±0.04335	0.08	Almond flavor
Phenol, 3,5-bis(1,1-dimethylethyl)-	0.14815±0.10243	0.20623±0.05978	0.04749±0.04238	-	-
Styrene	0.86720±0.87408	0.47409±0.47311	0.06142±0.11847	0.035	Tuberose fragrance
Benzene, 2-ethyl-1,4-dimethyl-	0.31038±0.34599	0.15266±0.06931	0.07448±0.10659	-	-
Butane dioic acid, phenyl-	1.44275±1.66138	0.62538±0.4585	0.16148±0.3764	-	-
1-Hydroxy-3-phenylacetone	0.00769±0.00733	0.00917±0.00461	0.01653±0.02855	-	-
Ethyl isovalerate	0.01211±0.01642	0.00216±0.00175	0.07410±0.06846	0.000013	Fruit flavor (apple, pineapple)
Hexanoic acid, 2-phenylethyl ester	0.05557±0.04613	0.09966±0.07632	0.02546±0.03591	-	Sweet honey scent

Propanoic acid, 2-phenylethyl ester	0.07079±0.057	0.10599±0.02086	0.04019±0.03617	-	Floral aroma, Fruity scent
Acetic acid ethenyl ester	5.79177±5.55393	1.04280±0.69212	3.16810±2.34207	-	-
Ethyl 2-methylpropionate	0.00692±0.00886	0.00322±0.00265	0.03619±0.04103	0.000022	Sweet wine fragrance
Pantolactone	0.00111±0.00091	0.02856±0.03978	0.00721±0.00904	-	Cotton candy
3-Octanone	0.11208±0.15112	0.11643±0.05692	0.06808±0.04772	0.0214	Fruit flavor
2-Propanone, 1-hydroxy-	0.12913±0.07242	0.08236±0.04257	0.17519±0.20093	80	Caramel aroma
3-Hexen-2-one, 5-methyl-	0.05590±0.10961	0.05033±0.0647	0.03640±0.04259	-	Fruity scent
2-Hexanone	0.17162±0.16704	0.33369±0.31097	0.40755±0.42527	0.56	Fruity scent
2-Butanone	2.08931±1.73966	3.41361±2.57363	7.13374±7.78654	35.4002	-
2,5-Hexanedione	0.14431±0.14825	0.38526±0.23966	0.92425±1.02918	-	-
Acetophenone	0.59591±0.50522	0.38088±0.10993	0.07793±0.11781	0.065	Pungent floral scent
5,9-Undecadien-2-one,6,10-dimethyl-	0.42187±0.27593	0.40279±0.12996	0.17565±0.1808	-	Fresh rose leaf floral scent
Pyrazine, methyl-	0.06085±0.04081	0.07473±0.04311	0.18783±0.21308	30	Nutty, baked aroma
Pyrazine, 2,6-dimethyl-	0.16672±0.20066	0.03514±0.04415	0.31680±0.82008	0.718	Cocoa, nut, roasted meat flavor
Diazene, dimethyl-	6.90739±6.02263	12.85098±3.42171	5.61519±3.67872	-	-
Pyrazine, trimethyl-	0.00876±0.00836	0.00931±0.00568	0.13739±0.33969	0.35012	Cocoa, baking aroma

Pyrrole	0.21214±0.17325	0.22945±0.08654	0.07779±0.0859	20	Nutty flavor
2-Pyrrolidinone, 1-methyl-	0.06111±0.03883	0.06255±0.03575	0.03243±0.03308	-	Slightly amine-like odor
Propanoic acid, 3-(methylthio)-	0.00359±0.00307	0.00242±0.00139	0.01965±0.04119	-	Sweet sulfur smell
S-Methyl propanethioate	0.00296±0.00327	0.00760±0.00537	0.06579±0.08362	-	Garlic flavor
S-Methyl 2-methylpropanethioate	0.05169±0.04098	0.33290±0.28575	1.83720±5.0503	-	Stimulating fruit flavor
Dimethyl sulfone	2.64021±1.44494	6.78305±3.92691	2.78258±3.44538	-	Sulfur smell
Furan, 2-methyl-	0.02130±0.03112	0.55755±1.22269	0.16521±0.41163	0.2	Chocolate flavor
2-Pentylfuran	0.20781±0.24128	0.17069±0.07978	0.04803±0.04588	0.0058	Fruity flavor
Furan, 2,5-dimethyl-	0.01838±0.01871	0.03417±0.01792	0.03148±0.03521	-	Roasted meat flavor
Indole	0.10808±0.10814	0.19288±0.12464	0.03825±0.03738	0.011	fecal

- Means no indicators or data are available. All results are presented as mean values (n = 8) ± standard error. TS, Taihe black-boned silky fowl; CB, crossbred black-boned silky fowl; HL, Hy-line Brown.
